# Effects of Modified Multistage Field Test on Performance and Physiological Responses in Wheelchair Basketball Players

**DOI:** 10.1155/2015/245378

**Published:** 2015-02-24

**Authors:** Thierry Weissland, Arnaud Faupin, Benoit Borel, Serge Berthoin, Pierre-Marie Leprêtre

**Affiliations:** ^1^Laboratoire de Recherche Adaptations Physiologiques à l'Exercice et Réadaptation à l'Effort, EA 3300, UFR-STAPS, Université de Picardie Jules Verne, 80000 Amiens, France; ^2^Laboratoire Motricité Humaine Education Sport Santé, EA 6312, UFR-STAPS, Université de Toulon, 83130 La Garde, France; ^3^LAMHESS, EA 6312, Université Nice Sophia Antipolis, 06205 Nice, France; ^4^Laboratoire Handicap, Activité, Vieillissement, Autonomie, Environnement, EA 6310, Département STAPS, Université de Limoges, 87060 Limoges, France; ^5^Equipe Activité Physique, Muscle, Santé, EA 4488, UDSL2, Université Lille Nord de France, 59000 Lille, France

## Abstract

A bioenergetical analysis of manoeuvrability and agility performance for wheelchair players is inexistent. It was aimed at comparing the physiological responses and performance obtained from the octagon multistage field test (MFT) and the modified condition in “8 form” (MFT-8). Sixteen trained wheelchair basketball players performed both tests in randomized condition. The levels performed (end-test score), peak values of oxygen uptake (VO_2peak_), minute ventilation (V_Epeak_), heart rate (HR_peak_), peak and relative blood lactate (Δ[Lact^−^] = peak – rest values), and the perceived rating exertion (RPE) were measured. MFT-8 induced higher VO_2peak_ and V_Epeak_ values compared to MFT (VO_2peak_: 2.5 ± 0.6 versus 2.3 ± 0.6 L·min^−1^ and V_Epeak_: 96.3 ± 29.1 versus 86.6 ± 23.4 L·min^−1^; *P* < 0.05) with no difference in other parameters. Significant relations between V_Epeak_ and end-test score were correlated for both field tests (*P* < 0.05). At exhaustion, MFT attained incompletely VO_2peak_ and V_Epeak_. Among experienced wheelchair players, MFT-8 had no effect on test performance but generates higher physiological responses than MFT. It could be explained by demands of wheelchair skills occurring in 8 form during the modified condition.

## 1. Introduction

A competitive wheelchair basketball game induced high cardiovascular stress [1–3]. It has been reported that wheeling tasks including sprint, endurance, and slalom were strongly correlated with aerobic fitness in wheelchair basketball players (WBP) [4, 5]. Schmid et al. [1] also showed that the higher the peak of oxygen uptake (VO_2peak_) was, the greater the game level of WBP was. Thus, assessment of aerobic fitness is essential for coaches or therapists to directly determine training or reconditioning intensities. To assess physiological and/or mechanical responses of wheelchair athletes or wheelchair-dependent persons, researchers and rehabilitation staff use continuous graded standardized protocols in laboratory such as arm crank exercises or wheelchair treadmill exercises [6–9]. However, results obtained during these tests are not sufficient for the coaches of wheelchair sports who prefer adapted wheelchair field tests because laboratory data are not related to the pushing proficiency, sport-specific skills techniques of wheeling, and individual adaptations to their wheelchair [3, 9]. Moreover, it has been shown that field tests induce significantly underestimated VO_2peak_ compared to VO_2peak_ measured during laboratory tests in the same subjects, irrespective of the disability [10, 11]. Difference in wheelchair configuration and specific propulsion required by field exercises could explain in part the previous results and thus the limitation of treadmill/ergometer evaluation to predict performance [4, 9]. Field exigencies may cause a peripheral muscular limitation, which does not allow achieving the maximum cardiorespiratory performance [11, 12]. However, evaluation of aerobic fitness component provides the physical preparation of wheelchair-dependent athletes with a possibility of improving the pushing performance and techniques of wheeling. The maximal aerobic velocity is a functional indicator of wheelchair-user combination and appears an interesting data to coaches or therapists as directly usable for determining training or reconditioning intensities. Previously, Vanderthommen et al. [13] proposed an incremental multistage field test (MFT) adapted to indoor practice. In a small space (17 m^2^), MTF indirectly estimated the maximal aerobic capacity according to the number of exercise levels (MFT-score) from the following equation: PeakVO_2_ = 18.03 + 0.78MFT-score [13].

They decided the accuracy and reliability of MFT to estimate VO_2peak_ and accessible protocol consisted to increase, in stages, the wheelchair velocity of the subject around an octagon in the same rotation direction as previously defined. In practice, turning around plots is very accessible for beginners because it demands little manoeuvrability and no initial acceleration other than a shuttle run [12], especially for people with motor impairments side. However, some criticisms would be advanced. Firstly, turning in the same direction could induce premature tiredness and muscle fatigue in the upper limb of the external curve. This could be in relation to the great push power output and high arm frequency in the synchronous mode of propulsion [14] and the centrifugal force exerted on the wheelchair in the curve. Secondly, performance in wheelchair sports relies not only on fitness but also on sport-specific skills, experience, and technical proficiency [9] like change in direction [11]. Vanlandewijck et al. [15] have been previously proposed a field-based drill test during one minute in an “8 form” around two cones positioned 5 meters from each other, to assess and agility performance for wheelchair basketball players. Changing the MFT test to describe “eight form” instead of a “round form” may distribute the forces of propulsion in both arms and reduce localized muscle fatigue while approaching movement forms encountered in the sport. This adaptation could allow assessing the performance to drive the wheelchair in both directions of rotation and request greater propulsive efficiency. Associating a continuous and progressive wheeling velocity to a succession of 8 turns could enable wheelchair users to exploit their mobility performance and thus achieve greater physiological response. This aspect could be of practical importance and potential application to the training and physiological assessment of wheelchair basketball players.

Thus, the objective of this study was to compare the physiological responses and the end-test scores obtained from the MFT with the modified condition in 8 (MFT-8). We hypothesized that MFT-8 induced a higher end-test score with higher cardiorespiratory responses due to a lesser local fatigue compared to the original MFT.

## 2. Materials and Methods

### 2.1. Subjects

The Paralympic Movement offers basketball opportunities for athletes that have variable upper-body skills due different impairments and injury level. For the international competition, players are placed into eight classification levels for participation (from 1 to 4.5) according to player's “volume of action,” based on the International Wheelchair Basketball Federation (IWBF) [16]. 16 trained wheelchair basketball players (WBP) were recruited (14 males and 2 females) and they reported 6.6 ± 2.3-year training duration. The population included participants with cerebral palsy (*n* = 2), spina bifida (*n* = 2), orthopedic impairments (*n* = 2), paraplegia (*n* = 6), above knee amputee (*n* = 2), lower limb agenesis (*n* = 1), and poliomyelitis (*n* = 1) (Table 1). Participants at this study represent seven classes of the eight IWBF based on the players' functional capacity to complete the skills necessary to play. All participants performed several training sessions per week and were engaged in national wheelchair basketball competitions every week. Two women were included because they belong to teams for several years called upon in this study. Skinfold thickness at 4 sites (triceps, subscapular, suprailiac, and abdominal) was measured using a Harpeden caliper.

For both tests, the players always used their own basketball wheelchair with the same adjustment (seating, wheels, or handrim). Before each test, the tyre pressure was checked with a gauge and the same optimal pressure (100% of the manufacturer's recommended level) was used at each time [17]. All subjects provided their written informed consent to participate in the study, which conformed to the recommendations of the Declaration of Helsinki and was approved by the local Ethical Committee.

### 2.2. Incremental Procedures

**Figure 1:**
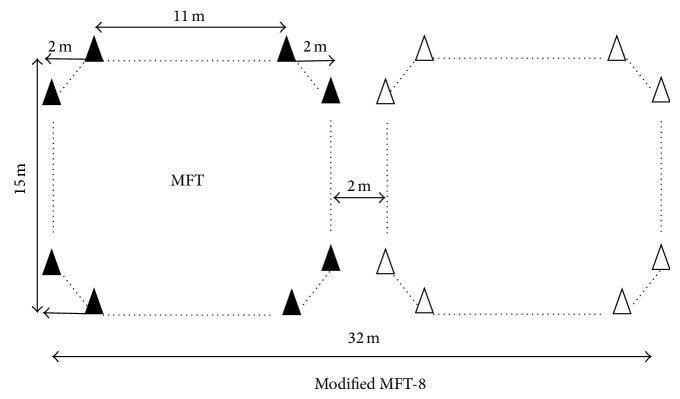
Illustration of MFT (full triangles track) and modified MFT (MFT-8, adding empty triangles track to MFT).

On two separate days, all subjects performed, in randomized order, in the same indoor hall with a hard surface (temperature ~18°C): (1) an incremental multistage field test (MFT) and (2) a modified MFT (MFT-8). The MFT consisted of wheeling around an octagonal course [13]. For both tests, the initial wheeling velocity was 6 km·h^−1^ for the first 1 min stage and then wheeling speed was increased by 0.37 km·h^−1^ every minute until exhaustion. With the same progressive velocity, the MFT-8 consisted of turning around two octagons distant to 2 meters to describe an 8 (Figure 1). MFT-8 needed a larger surface (MFT-8: 32 × 15 m versus MFT: 15 × 15 m) while respecting the same instructions from auditory feedback as the original MFT. In practice 4 subjects can be assessed simultaneously with MFT-8. Both tests were stopped when the subject could no longer be located within the turning zone at beep signal despite verbal encouragements. Therefore, MFT and MFT-8 end-test scores were the longer time maintaining the speed imposed on the stage (time to exhaustion) during each respective test. All subjects were advised to maintain a regular diet and drink during the day before testing (keeping the same meals) and to refrain from smoking and caffeinated drinks during the two hours prior to testing.

### 2.3. Physiological and Perceived Responses Measurements

Oxygen uptake (VO_2_), carbon dioxide production (VCO_2_), breathing frequency (RF), and minute ventilation (V_E_) were measured breath-by-breath at rest and throughout both tests using Cosmed K4b² or Metamax 3B ambulatory systems. To minimize the turnover of subjects, two portable measurement systems were used. To repeat the mistake device, the subjects always used the same analyzer for both tests. A previous study showed a satisfactory comparison between the two measuring devices with cyclists [18]. The turbines flow volume was calibrated by using a 3 L syringe and the analysers were calibrated before each test, according to the constructor instruction manuals using ambient air and calibration gas (16% O_2_ and 5% CO_2_). Then we used the software of each device to automatically eliminate ectopic values and average the data every 5 seconds. In addition, heart rate (HR) was recorded beat-to-beat and averaged every 5 seconds. The estimated VO_2_ was calculated by Vanderthommen's formula [13].

Capillary blood samples (25 *μ*L) for [Lact^−^] were collected from the finger at rest ([Lact^−^]_rest_) to assess basal blood lactate concentration, immediately after cessation of the test and after 3 min of the passive recovery. Peak [Lact^−^] and relative (Δ[Lact^−^] = peak[Lact^−^] − [Lact^−^]_rest_  values) blood lactate values were used for analysis. All blood samples were analyzed using a portable lactate analyzer (Lactate Pro, Arkray, Inc. Kyoto, Japan).

Immediately after the end of test, participants reported their rating of perceived exertion (RPE) using the 6–20 Borg scale [19]. RPE values were classified according to the American College of Sports Medicine stand as very light (<9), light (9–11), moderate (12-13), vigorous (14–17), and near maximal to maximal (≥18). Participants were familiar with prior study to use the scale RPE.

### 2.4. Statistical Analysis

Descriptive data are presented as mean standard deviation (mean ± SD) and 95% confidence intervals. Normality and homogeneity of the distribution were verified via Shapiro-Wilks and Levene tests, respectively. Student's *t*-tests were used to compare the resting, time to exhaustion, end-test score, and physiological peak values measured during MFT and MFT-8 exercises. Pearson's correlation coefficients (*R*) were used to examine the relationships between end-test score, VO_2peak_, V_Epeak_, and condition test. The magnitude of effects was qualitatively assessed according to Hopkins et al. [20] as follows: *r* < 0.1, trivial; 0.1–0.3, small; 0.3–0.5, moderate; 0.5–0.7, large; 0.7–0.9, very large; >0.9, nearly perfect; and 1.0, perfect. In all statistical analyses, the (alpha) level of significance was set at *P* < 0.05.

## 3. Results

**Figure 2:**
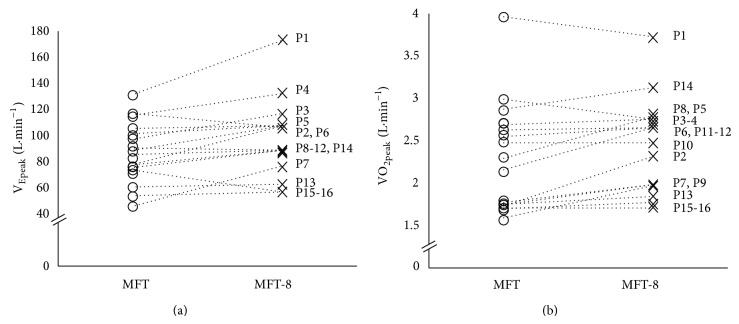
(a) Peak ventilation (V_Epeak_ in L·min^−1^) and (b) oxygen uptake peak values (VO_2peak_ in L·min^−1^) per basketball wheelchair players (P, *n* = 16) measured during classic (MFT, open circle) and modified (MFT-8, cross) multistage field test.

**Figure 3:**
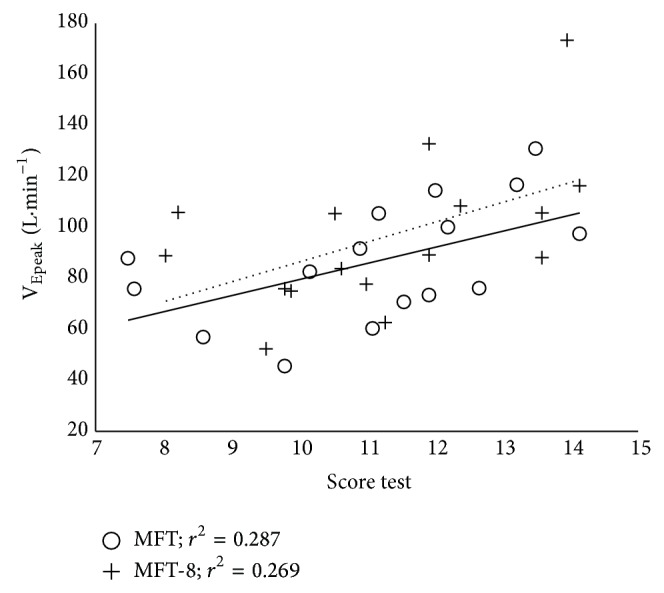
Relation between peak of pulmonary ventilation (V_Epeak_ in L·min^−1^) and end-score test values measured during classic (MFT, open circle) and modified (MFT-8, cross) multistage field test in basketball wheelchair players (*n* = 16). Continuous line represented the linear regression for MFT (*r* = 0.54, *R* ^2^ = 0.287, *P* = 0.03), the dash line, and the linear regression for MFT-8 (*r* = 0.52, *R* ^2^ = 0.269, *P* = 0.04).

The sample of 16 wheelchair basketball players was heterogeneous for physical characteristics and impairment defined by the International Wheelchair Basketball Federation classification (Table 1). We found similar time to exhaustion between both tests (14 min 51 s ± 5 min 22 s for MFT and 15 min 15 s ± 5 min 13 s for MFT-8; *P* > 0.05). Individual peak ventilation and oxygen uptake per wheelchair player connected from MFT and MFT-8 were reported in Figure 2. Peak oxygen uptake and ventilation were higher at the end of MFT-8 compared to MFT (VO_2peak_:  2.5 ± 0.6 versus 2.3 ± 0.6 L·min^−1^ and V_Epeak_:  96.3 ± 29.1 versus 86.6 ± 23.4 L·min^−1^, *P* < 0.05, resp.) (Table 2), while the mean end-test score, peak RF, peak HR, Δ[Lact^−^], peak [Lact^−^], and peak RPE showed no significant difference between MFT and MFT-8 tests. In both tests, end-test scores were significant related to V_Epeak_ (*r* = 0.54 for MFT and *r* = 0.52 for MFT-8, *P* < 0.05; Figure 3) but not with VO_2peak_. End-test score and VO_2peak_ were related with condition test (*r* = 0.93, *P* < 0.001 for end-test score and *r* = 0.84, *P* < 0.001 for  VO_2peak_ values).

No significant difference was found between peakVO_2_ estimated by Vanderthommen regression and measured VO_2peak_ for MFT (29.6 ± 4.2 mL·min·kg^−1^ versus 32.1 ± 7.8 mL·min·kg^−1^). No variable was correlated with the impairment defined by IWBF classification except V_Epeak_ (*R* = 0.57, *P* < 0.05).

## 4. Discussion

### 4.1. Comparison between MFT and MFT-8

The original aim of this study was to compare the field test score and physiological responses in trained wheelchair basketball players engaged in two ramp exercises: an octagonal figure (MFT) and an “8” figure (MFT-8). The main findings reveal that end-test scores, HR, RPE, and blood lactate peak values were not affected by changes in direction but VO_2peak_ and V_Epeak_ were significantly higher in MFT-8 than MFT (*P* < 0.05). The end-test score was also correlated between both tests (*R* = 0.93, *P* < 0.001). Generally, the attainment of peak values of physiological responses was influenced by the testing procedure (initial workload, ramp rate, time stage, and crank rate) and by the level of physical capacity [7]. In active subjects, a ramp with a small increment underestimated the arm power output without significant change in peak of HR and VO_2_ values [1]. The increase in respiratory responses to MTF-8 without significant change in other parameters cannot be attributed to increase in velocity between each stage while it was the same for both tests. In contrast to MFT in which the participants determine their preferred choice of rotation, shaped profile in 8 did not offer this possibility. This condition disadvantages the athletes with disabilities who often have a functional asymmetry with a dominant side and contralateral side deficit in strength, balance, sensibility, and coordination [11]. Improving the push technique and mobility performance acquired by wheelchair basketball skills may have reduced the disturbances imposed by changes in direction of MFT-8 without significant effect of time to exhaustion.

The “8” figure should lessen the monotonous perception to turn in round and promote the asynchronous mode of propulsion and aimed to reduce possible asymmetry muscular fatigue. In the present study, we observed that the use of lateral and contralateral sides had a significant impact on both peak oxygen uptake and ventilation responses. The initial assumption that performance output was greater than the MFT-8 is dismissed from the mechanical point but accepted in terms of physiological responses. We also found for both tests a significant relationship between V_Epeak_ and the end-test score but not with VO_2peak_. A significant alteration in respiratory function is a characteristic among subjects with abdominal and intercostal muscles paralysis or weakness and V_Epeak_ could be more sensitive than VO_2peak_ [7, 21, 22]. The increase in V_E_ without RF significant variation can be explained by an improvement in tidal volume achieved by the mode of the propulsion in wheelchair player. The agility to apply the force on the hand-rim and to change direction quickly needed in the MFT-8 contributes to increased oxygen uptake and volume expired. However, a standardized test ergometer would have to compare and verify the completeness of VO_2_ and V_E_ peak values measured at MFT-8. Vanderthommen et al. [13] had not standardized physiological responses with a reference test on ergometer. Thus, the MFT-8 has the advantage of assessing, in addition to physical fitness, the technical performance to maintain wheeling velocity when succession of alternating turns. For heterogeneous people trained as wheelchair basketball players, MFT-8 did not affect the times of exhaustion or end-test scores; it would be useful to verify it in nonathletic people or in rehabilitation with a poor wheelchair experience.

With important group of subjects, we would tend to focus on the conventional protocol due to its compactness, because in the same place, the assessment of a team is achieved faster with the MFT (grouping of 8 players while only 4 for MFT-8).

### 4.2. MFT Responses

Field tests can explore the physiological fitness associated with ability of a wheelchair displacement and provide practical uses for wheelchair athletes. Continuous [23, 24] and shuttle [11, 15] adapted wheelchair field tests have been proposed to estimate performance and maximal aerobic speed in order to predict VO_2peak_. For the sample of 16 players, means MFT scores measured were higher than those of Vanderthommen's sample values (14.9 ± 5.1 score versus 9.2 ± 5.8), as well as VO_2peak_ (32.1 ± 7.8 versus 25.2 ± 5.9 mL·min·kg^−1^) and peak [Lact^−^] (8.6 ± 3.2 versus 5.4 ± 1.9 mmol·L^−1^) but with a similar HR_peak_ (173.0 ± 14.4 versus 172.0 ± 5.9 b·min^−1^) [13]. Several points can explain these differences. Firstly, the training levels of the subjects were included in the current studies because they consisted of WBP while Vanderthommen's subjects were active but not licensed in any disabled sport federation. Training exercise increases not only the aerobic and anaerobic capacities but also the efficiency of wheelchair propulsion [1, 9, 12]. Secondly, using material of specific basketball wheelchair, due to its rigidity, lightweight, and camber, allows greater rotation facilitates. Hilbers and White [25] found that the wheelchair design has a very significant influence on the energy used in propulsion compared to conventional chairs. Beekman et al. [26] showed that the use of lightweight wheelchairs increased the speed and distance and decreased oxygen consumption (at a same velocity) in people with spinal cord injury on outside tracks. Finally, the combination of factors involving the user, the wheelchair, and their interaction contributes to a better optimization of the performance of mobility. Mason et al. [27] showed that various adjustments of the wheelchair (rear wheel camber, wheel size, seat positioning, hand-rim configuration, and fore-aft seat position) depending on the physiological and biomechanical characteristics of wheelchair user are determining optimal performance in wheelchair court sports. In the present study, the interaction between the wheelchair and the user erases the performance output. The therapist must take into account all aspects for patients during rehabilitation regularly trying different settings and wheelchairs before the final selection. With a mean experience of 6.5 years of basketball practice, our subjects optimized the ergonomics of their wheelchair compared with untrained wheelchair patients of the study of Vanderthommen [13]. Hence, in trained athletes, the MFT score is not directly related to a limit on the cardiorespiratory function but rather a muscle and functional potential, while for MFT-8, V_Epeak_, and VO_2peak_ values tend to show the opposite.

In a field assessment of paralympic athletes with the similar average IWBF level (2.9 ± 1.3) HR_peak_ and VO_2peak_ values (174.4 ± 11.6 b·min^−1^ and 34.1 ± 4.2 mL·min^−1^·kg^−1^), Bernardi et al. [3] observed a significant relationship between oxygen consumption obtained in the field and that measured in the laboratory during an arm crank exercise (ACE). The latter also reported lower blood lactate values in field condition compared to ACE condition (3.9 ± 0.9 versus 11.10 ± 1.92 mmol·L^−1^, *P* < 0.05). They explained this difference by the intermittent nature of the exercise of the field test. In the present study, high values of [Lact^−^]_peak_ were observed (8.6 ± 3.2 mmol·L^−1^) which could be due to the design of multistage field test comparable to the incremental ACE laboratory test.

We found no significant difference between the mean VO_2peak_ measured during MFT test for all population and estimated PeakVO_2_ [13] with end-test score by the predictive equation (32.1 ± 7.8 versus 29.6 ± 4.2 mL·min^−1^·kg^−1^). Good estimate of PeakVO_2_ by MFT end-score despite the level difference, heterogeneity of subjects, and floor surface is a support for coaches to estimate oxygen consumption of players regardless of their classification IWBF. However, by expressing the values of VO_2peak_ according to the score we do not obtain significant relationship as opposed to setting V_Epeak_ (Figure 3). The therapists will also consider this correlation if they are required to test the maximum cardiorespiratory system of patients.

Goosey-Tolfrey et al. [6] who assessed separated wheelchair athletes with or without trunk stability and balance classification found a significant difference between both groups of VO_2peak_ (absolute and relative). More recently, these authors have shown a decreasing relationship between peak power output achieved in a Wingate test and sporting classification [9]. In relation with wheelchair classification, de Lira et al. [8] demonstrated that the functional classification score was positively correlated with oxygen uptake (peak and at ventilatory threshold) and anaerobic parameters in wheelchair basketball players ranged from class 1.0 to 4.5 without any relationship with the peak treadmill velocity (*r* = −0.14, *P* = 0.59). Skill tests in wheelchair basketball showed significant performance differences between opposite functional classes (low-level lesion and high-level lesion or cerebral palsy) with similar performance between the low classes represented by 1.0 to 2.0 points but reduced with minimal disability such as in classes 2.5 through 4.5 [28]. However, in a recent study, Yanci et al. [29] demonstrated the good reliability of intermittent aerobic test and agility *t*-test in wheelchair basketball players without any significant inference of classification.

The most exhaustive MFT-8 could let understand that wheeling around single direction in MFT has a reducing effect of residual physical capacities of subjects with less impairment.

Practical recommendations for a basketball team would be to use the MFT-8 for all players without distinguishing between IWBF classifications to improve their rolling speed in continuous exercises. In addition, to be closer to the requirements of wheelchair basketball, it might be interesting to compare aerobic fitness in the MFT and that measured from an intermittent field test.

## 5. Conclusions

The MFT-8 condition induced higher values of peak oxygen uptake and ventilation for same performance than MFT. Agility described by a succession of alternate turns and management of centrifugal and centripetal forces represents an increase of oxygen consumption and ventilation for wheelchair basketball player greater but without change of time of exhaustion and end-test scores. A strong significant relationship was found between the ventilation responses and performance according to the international classification while other measured parameters were not connected. Heterogeneity of pathologies, residual functional capacity and impairment impact on the physiological functions certainly are explanatory causes. For wheelchair-sports coaches and therapists, MFT-8 would be a tool combining the mobility performance and assessment of aerobic fitness.

## Figures and Tables

**Table 1:** Means ± SD and individual wheelchair basketball players' characteristics (gender, age, sum of four skinfolds: the biceps, triceps, subscapular, and suprailiac, disability, and wheelchair basketball playing experience) grouped according to International Wheelchair Basketball Federation classification (IWBF) [16].

Player	Sex	Age (years)	ΣSK (mm)	Disability	IWBF classification	Experience (years)
P1	M	31	46.4	Orthopedic impairments	4.5	6
P2	F	38	34.1	Above knee amputation	4.5	9
P3	M	30	56.9	Above knee amputation	4	2
P4	M	27	23.1	Cerebral palsy	4	6
P5	M	36	55.5	Spina bifida	4	9
P6	M	36	48.3	Spinal cord injury	4	8
P7	M	29	44.1	Cerebral palsy	3	5
P8	M	23	37.9	Spinal cord injury	3	7
P9	M	39	39.1	Spinal cord injury	3	7
P10	M	36	44.9	Agenesis	3	5
P11	M	39	55.5	Spina bifida	2.5	10
P12	M	41	27.2	Spinal cord injury	2	6
P13	M	30	42.9	Spinal cord injury	2	6
P14	M	27	52.2	Spinal cord injury	1.5	7
P15	F	28	76.2	Lower limb agenesis	1	4
P16	M	29	45.1	Poliomyelitis	1	7

Mean ± SD		32.4 ± 5.3	42.3 ± 13		2.9 ± 1.2	6.5 ± 2.3

**Table 2:** Means ± SD and 95% confidence interval (CI) of peak physiological values and end-test score measured during classic (MFT) and modified (MFT-8) multistage field tests in basketball wheelchair players (*n* = 16).

	End-test score	Peak RF	Peak V_E_	PeakVO_2_	Peak HR	Peak [Lact^−^]	Δ[Lact^−^]	Peak RPE
		b·min^−1^	L·min^−1^	L·min^−1^	b·min^−1^	mmol·L^−1^	mmol·L^−1^	
MFT	14.8 ± 5.4	48.8 ± 9.7	86.3 ± 23.7	2.3 ± 0.6	175.4 ± 12.5	8.6 ± 3.2	7.2 ± 3.4	15.3 ± 3.6
CI	*12.2–17.5 *	*44.1–53.6 *	*74.6–97.9 *	*1.9–2.5 *	*169.3–181.6 *	*7.0–10.2 *	*5.3–8.7 *	*13.6–17.2 *
MFT-8	15.3 ± 5.2	52.0 ± 11.9	96.3 ± 29.5^a1^	2.5 ± 0.6^a2^	173.3 ± 13.1	8.8 ± 3.0	6.7 ± 3.2	15.9 ± 3.1
CI	*12.7–17.8 *	*47.3–58.9 *	*81.8–110.8 *	*2.2–2.8 *	*166.9–179.7 *	*7.3–10.2 *	*5.1–8.2 *	*14.4–17.5 *

Breath frequency (RF), ventilation (V_E_), oxygen uptake (VO_2_), heart rate (HR), blood lactate ([Lact^−^]), and rating of perceived exertion (RPE). Δ[Lact^−^] represented the difference between rest and maximal blood lactate value measured during the exercise recovery.

^a^Significantly different from MFT (*P* < 0.05); ^1^moderate effect; ^2^small effect.
